# Novel Mannitol-Based Small Molecules for Inhibiting Aggregation of α-Synuclein Amyloids in Parkinson's Disease

**DOI:** 10.3389/fmolb.2019.00016

**Published:** 2019-03-22

**Authors:** Ashim Paul, Bo-Dou Zhang, Satabdee Mohapatra, Gao Li, Yan-Mei Li, Ehud Gazit, Daniel Segal

**Affiliations:** ^1^School of Molecular Microbiology and Biotechnology, Tel Aviv University, Tel Aviv, Israel; ^2^Department of Chemistry, Tsinghua University, Beijing, China; ^3^Institute of Parkinson Disease, Beijing Institute for Brain Disorders, Beijing, China; ^4^Center for Synthetic and Systems Biology, Tsinghua University, Beijing, China; ^5^Sagol Interdisciplinary School of Neurosciences, Tel Aviv University, Tel Aviv, Israel

**Keywords:** α-synuclein, Parkinson's disease, mannitol, NQTrp, aggregation, inhibitor

## Abstract

The aggregation of the amyloidogenic protein α-synuclein (α-Syn) into toxic oligomers and mature fibrils is the major pathological hallmark of Parkinson's disease (PD). Small molecules that inhibit α-Syn aggregation thus may be useful therapeutics for PD. Mannitol and naphthoquinone-tryptophan (NQTrp) have been shown in the past to inhibit α-Syn aggregation by different mechanisms. Herein, we tested whether the conjugation of Mannitol and NQTrp may result in enhance efficacy toward α-Syn. The molecules were conjugated either by a click linker or via a PEG linker. The effect of the conjugate molecules on α-Syn aggregation *in vitro* was monitored using Thioflavin T fluorescence assay, circular dichroism, transmission electron microscopy, and Congo red birefringence assay. One of the conjugate molecules was found to be more effective than the two parent molecules and as effective as a mixture of the two. The conjugate molecules attenuated the disruptive effect of α-Syn on artificial membrane of Large Unilamellar Vesicles as monitored by dye leakage assay. The conjugates were found to be have low cytotoxicity and reduced toxicity of α-Syn toward SH-SY5Y neuroblastoma cells. These novel designed entities can be attractive scaffold for the development of therapeutic agents for PD.

## Introduction

Intracellular or extra cellular deposits of amyloid aggregates of certain proteins are hallmarks of various neurodegenerative diseases (Ross and Poirier, 2004; Chiti and Dobson, 2006; Treusch et al., 2009). While, the mechanism of amyloid formation remains elusive, growing evidence suggests that the process proceeds through multiple steps including protein misfolding followed by formation of toxic oligomers and protofilaments, culminating into mature fibrils (Ross and Poirier, 2004; Stefani, 2004; Chiti and Dobson, 2006; Glabe, 2006; Knowles et al., 2014). α-synuclein (α-Syn) is such an amyloidogenic protein, whose toxic deposits in dopaminergic neurons in the brain, are involved in the pathogenesis of Parkinson's disease (PD) and Lewy body dementia (Spillantini et al., 1997; Conway et al., 2000; Dauer and Przedborski, 2003; Cookson, 2005; Bridi and Hirth, 2018; Ghiglieri et al., 2018). α-Syn is an intrinsically disordered protein which acquires a broad conformational diversity, presumably endowing it with the ability to play multiple functions (Mizuno et al., 2012; Wang et al., 2016). Yet, the exact role of α-Syn at physiological and pathological conditions is not fully understood (Bendor et al., 2013; Ghosh et al., 2015; Dettmer, 2018). An attractive therapeutic strategy toward synucleopathies would be to reduce formation of toxic oligomers and fibrils of α-Syn. Various attempts have been made along these lines, including the use of β-synuclein and its fragments (Shaltiel-Karyo et al., 2010; Leitao et al., 2018; Williams et al., 2018), nanobodies (Butler et al., 2016; Iljina et al., 2017), peptides (Madine et al., 2008; Choi et al., 2011, 2018), chaperones (Dedmon et al., 2005; Zhang et al., 2011), polydopamine dendrimers (Milowska et al., 2011), molecular tweezer (Prabhudesai et al., 2012), metal chelation (Mounsey and Teismann, 2012; Finkelstein et al., 2017), and various natural and synthetic small molecules (Zhu et al., 2004; Kobayashi et al., 2006; Masuda et al., 2006; Bieschke et al., 2010; Bisaglia et al., 2010; Meng et al., 2010; Scherzer-Attali et al., 2012; Singh et al., 2013; Ardah et al., 2014; Pujols et al., 2018), yet no such therapeutic is currently available. The various agents used exhibit certain limitations, including degradation by proteases and inefficient crossing of the blood brain barrier (BBB) (Begley, 2004; Werle and Bernkop-Schnürch, 2006; Gabathuler, 2010).

**Figure 1:**
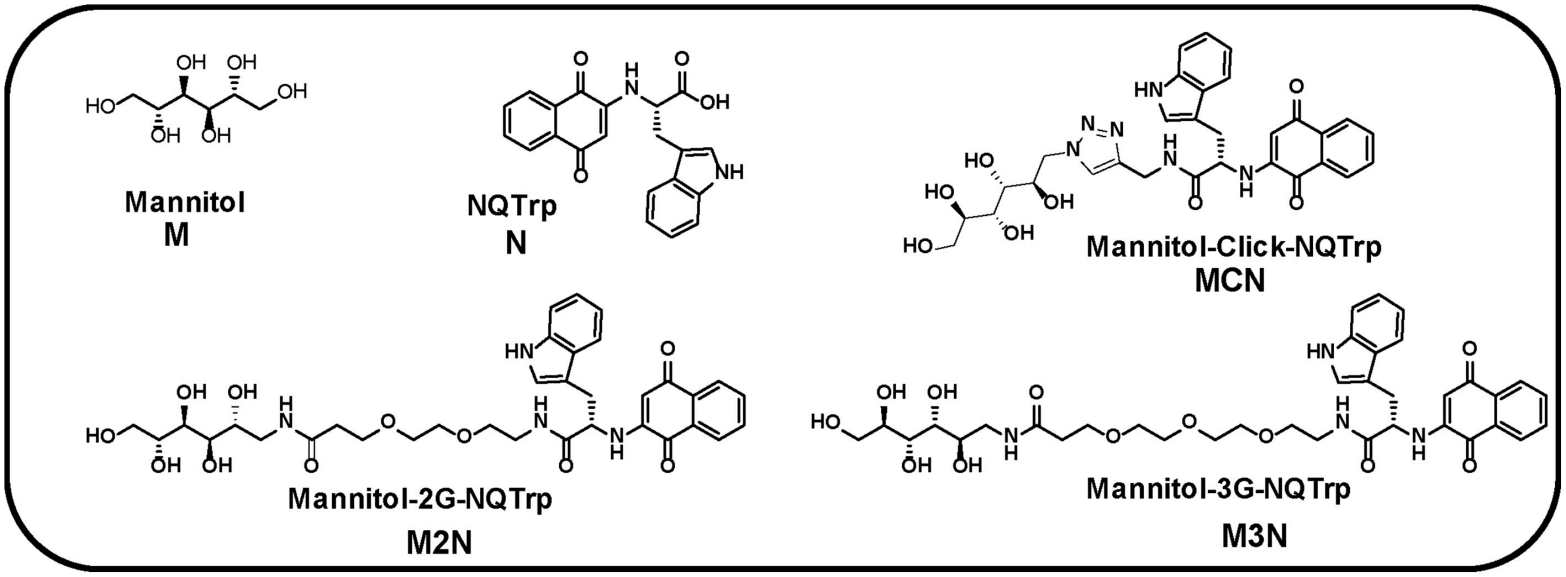
Chemical structures of Mannitol (**M**), NQTrp (**N**), and their derivative three conjugate molecules (**MCN**, **M2N**, and **M3N**). 2G and 3G indicate two and three PEG, respectively.

We have previously demonstrated inhibition of the self-assembly of α-Syn by Mannitol (**M**, Figure 1) which was found to act *in vitro* as a chemical chaperone stabilizing α-Syn structure through non-specific, solvent-mediated interactions (Shaltiel-Karyo et al., 2013). Administration of Mannitol intraperitoneally to transgenic mice expressing human α-Syn reduced its deposites in the brain accompanied by ameliotration of various PD pathologies (Shaltiel-Karyo et al., 2013). Notably, due to its hyperosmotic capacity, Mannitol is used clinically for disrupting the blood-brain barrier (BBB) and increasing its permeability to drugs (Suzuki et al., 1985; Pan et al., 2000). Thus a dual mechanism was suggested for the compound (Shaltiel-Karyo et al., 2013). Yet, for achieving inhibition of ~90% aggregation of α-Syn (100 μM) *in vitro*, a higher dose of Mannitol (0.225 M) was required, which can be a restriction from therapeutic perspective. In a separate series of experiments, we identified a quinone based derivative, Naphthoquinone-Tryptophan (NQTrp), as a generic inhibitor of fibril formation of various amyloidogenic proteins, which ameliorated Aβ- and tau-engendered symptoms in animal models (Scherzer-Attali et al., 2010; Frenkel-Pinter et al., 2016). The NQTrp molecule (**N**, Figure 1) was developed based on the established key role of aromatic residues in facilitating the recognition and self-assembly of the amyloid monomers and in stabilizing the resultant nano-fibrils (Gazit, 2002; Scherzer-Attali et al., 2010). By interfering with π-π stacking of α-Syn self-assembly, NQTrp inhibited its aggregation even at low concentration (2 mM), resulted in ~80% inhibition of α-Syn (100 μM) aggregation (Scherzer-Attali et al., 2012). A possible limitation of NQTrp from a drug development perspective is its restricted BBB permeability.

We hypothesized that by covalently linking NQTrp to Mannitol, its ability to cross the BBB may be enhanced. As a first step in this direction we examined here whether the conjugation of Mannitol and NQTrp would result in synergistic effect of the two parent molecules thus improving their potential efficacy toward α-Syn in PD. To that end we generated novel compounds in which these two small molecules are conjugated to each other either directly by click linker (Rostovtsev et al., 2002; Tornøe et al., 2002) or via a PEG linker. Using various biophysical assays, the conjugate molecules were found to delay the kinetics of α-Syn aggregation and to reduce the extent of the fibrils formed. In addition, the conjugates were non-toxic to cells and could reduce the cytotoxicity of α-Syn aggregates. The conjugates were more potent than either Mannitol or NQTrp or their mixture.

## Materials and Methods

Mannitol and NQTrp were purchased from Sigma-Aldrich (Rehovot, Israel). Compound **MCN**, **M2N**, and **M3N** (Figure 1) were synthesized by us as described below. Recombinant wild type α-Syn was purchased from rPeptide, USA (Catalog # S-1001-2). All lipids were purchased from Avanti® Polar Lipids (USA). Unless otherwise stated, all chemicals were obtained from Sigma-Aldrich (Rehovot, Israel and Innochem, Aladdin), and were of analytical grade.

## Synthesis of the Mannitol-NQTrp Conjugate molecules (MCN, M2N, and M3N)

### Synthesis of Compound MCN

The stepwise chemical synthesis of the compound **MCN** (Figure 1) is shown in Scheme S1.

For the synthesis of compound **MCN**, first compound **2** (Scheme S1) was prepared using the following protocol. 2-Propynylamine (38 mg, 0.417 mmol) was dissolved in 1 mL THF/DCM (7/3) solvent. Then, EDC•HCl (79.7 mg, 0.4 mmol), HOBt (41 mg, 0.3 mmol), and DIEA (200 μL) were added into the solution followed by dropwise addition of 100 mg (0.28 mmol) compound 1 (Scheme S1) dissolved in 100 μL THF. After 12 h, the solution was dried under N_2_ and the compound **2** (Scheme S1) was purified by column chromatography (cyclohexane/ethyl acetate = 4/1) with yield of 91%. Compound **2** was characterized by mass spectrometry (Figure S1).

Next, compound **6** (Scheme S1) was synthesized as follows. D-Mannitol (10 g, 54 mmol) and 100 mg DMAP (4-dimethylaminopyridine) were dissolved in 80 mL pyridine. Then, 4-toluene sulfonyl chloride (TsCl, 7.8 g, 41 mmol) in 80 mL pyridine was added dropwise into D-mannitol solution at 0°C with stirring. After 24 h, 62 mL of Ac_2_O were added dropwise into the above solution at 0°C. After additional 24 h, the reaction was quenched with water and the crude organic product was collected after acid-base wash. The crude product was resolved by column chromatography (cyclohexane/ethyl acetate = 4/1) to obtain compound **5** (Scheme S1), which further reacted with NaN_3_ in DMF at 80°C for 24 h to obtain compound **6**. The product was separated by column chromatography (cyclohexane/ethyl acetate = 9/1) to obtain compound **6**. Then, compound **6** (0.151 mmol) in 0.15 mL H_2_O/CH_3_CN (2/1) and compound **2** (0.125 mmol) in 0.5 mL H_2_O/CH_3_CN/THF (1/2/3) were mixed in presence of sodium ascorbate (0.126 mmol) in 0.1 mL H_2_O and CuSO_4_ (0.0126 mmol) in 0.05 mL H_2_O, for 12 h. Then, the mixture was purified by semi-preparative HPLC using C18 column with linear gradients of 40% to 90% of solution B (80% CH_3_CN/H_2_O with 0.06% TFA) in solution A (H_2_O with 0.06% TFA) for 30 min (λ = 215 nm). After lyophilization, 43.3 mg of compound **7** (Scheme S1) was obtained with yield of 42%. Compound 7 was characterized by mass spectrometry (Figure S2).

For synthesis of compound **MCN**, 13.3 mg of compound **7** (Scheme S1) were dissolved in 3 mL CH_3_OH. CH_3_ONa was used to adjust the pH of solution to ~8.5. After 19 h, the reaction was neutralized with AcOH and the solvent was removed under vacuum. The product was dissolved in 6 ml CH_3_CN and purified with semi-preparative HPLC using C18 column with linear gradients of 30% to 80% of solution B (80% CH_3_CN/H_2_O with 0.06% TFA) in solution A (H_2_O with 0.06% TFA) for 30 min (λ = 215 nm). After lyophilization, 8 mg of compound **MCN** was obtained with yield of 82%. The purified compound was further characterized by HPLC (Figure S3) and mass spectrometry (Figure S4).

### Synthesis of Compounds M2N and M3N

The stepwise chemical synthesis of the compounds **M2N** and **M3N** is shown in Scheme S2.

A solution of 1-Amino-1-deoxy-D-mannitol (Peterson et al., 2011) (1.60 mmol) in DMSO (1 mL) was added drop wise to a solution of DIEA (6.40 mmol), linker (1.92 mmol), HOBT (2.40 mmol), and EDC-HCl (2.40 mmol) in 4 mL DMSO at 0°C for 10 min. The mixture was stirred at room temperature for 12 h and 40 mL water were added and washed with CH_2_Cl_2_ (4 × 20 mL). The water layer was lyophilized to obtain a white powder. The white powder was added to 3 mL 20% piperidine/DMSO solution and was stirred for 1 h at room temperature. The reaction mixture was dropped into 40 mL of cold ether to obtain light yellow precipitate of linker-saccharide conjugate, which was dried under vacuum. Next, NQTrp (0.138 mmol) (Shrestha-Dawadi et al., 1996), DIEA (0.552 mmol), HOBT (0.207 mmol), and EDC-HCl (0.207 mmol) were dissolved in 3 mL DMSO and kept at 0°C. After 10 min, the reaction mixture was added to linker-saccharide conjugate solution (0.138 mmol) in 1 mL DMSO, and the mixture was stirred at room temperature for 12 h. The mixture was dropped into 40 mL of cold ether to obtain orange precipitate, which was dissolved in 2 mL CH_2_Cl_2_ and purified by flash column chromatography to obtain the final compound **M2N**/**M3N**. Compound **M2N** (*n* = 2, Scheme S2) and **M3N** (*n* = 3, Scheme S2) were further characterized by HPLC and mass spectrometry (Figures S5–S8).

### Liquid Chromatography

Purity of the compounds (**MCN**, **M2N**, and **M3N**) was confirmed by Waters UPLC-MS system (ESI). Solvents used: solvent A (0.1% formic acid in H_2_O) and solvent B (0.1% formic acid in CH_3_CN) on C18 ultra analytical column with a flow rate of 0.5 mL/min. Dual wavelength were selected at 220 and 280 nm. Linear gradient of 5–95% CH_3_CN was used in a total run time of 10 min.

### Mass Spectrometry

Masses of the purified samples were analyzed on Waters UPLC-MS (ESI +ve mode) Micromass Q-TOF equipped with Masslynx software.

### Stock Preparation

α-Syn was monomerized by a 10 min pretreatment with HFIP and the solvent was evaporated using a Speed Vac. The resulting thin film was dissolved in phosphate buffer saline (PBS) and sonicated for 5 min. The working buffer system for all assays was PBS (100 mM, pH 7.4). Concentration of the protein was determined using Nano drop (calculated according to ε_280_ of 1490 M^−1^cm^−1^) and adjusted to 50 μM concentration as a stock solution. Stock solutions of Thioflavin T (ThT, 4 mM) was prepared in 100 mM PBS. Stock solution hybrid molecules (10 mM) were prepared separately in DMSO and diluted with PBS before use.

### Thioflavin T Assay

For monitoring aggregation kinetics of α-Syn, the stock solutions were diluted in 100 μL wells in a 96-well black plate so that the final mixture contained 10 μM of the protein and 20 μM ThT in 100 mM PBS. The inhibitor molecules (at 5:1, 1:1, 1:5 ratio of α-Syn:inhibitor molecule) were added separately to designated wells and kinetics of α-Syn aggregation was monitored by ThT fluorescence at 37°C with continuous shaking. The data were collected (in triplicate manner) using Infinite M200 microplate reader (Tecan, Switzerland), with measurements taken at 15 min intervals for 50 h. Excitation and emission wavelengths of ThT were 440 and 485 nm, respectively.

### Circular Dichroism Spectroscopy

To analyze the secondary structure of the inhibited α-Syn, 300 μL of the samples were taken in a cuvette (path length 1 mm) and CD spectra were then recorded on a Chirascan spectrometer between the range of 190–260 nm, and the background was subtracted from the CD spectra. Since, DMSO absorbs at far UV range, stocks of hybrid molecules were prepared in methanol for this assay.

### Transmission Electron Microscopy

Samples (10 μL) were placed for 2 min on 400-mesh copper grids covered with carbon-stabilized Formvar film (Electron Microscopy Sciences, Hatfield, PA). Excess fluid was removed, and the grids were negatively stained with 2% uranyl acetate solution (10 μL) for 2 min. Then, the excess fluid was removed and allowed to dry for 5 min. The samples were viewed using a JEM-1400 TEM (JEOL), operated at 80 kV.

### Congo Red Birefringence

Congo red powder was dissolved in 80% aqueous ethanol to prepare a saturated stock solution. The aggregated α-Syn solution (5 μL) in the absence or presence of different doses of the inhibitors were mixed with 5 μL of saturated Congo red solution. The suspension was drop casted over a glass side and the samples were dried in air and kept in a desiccator before birefringence analysis. The samples were viewed at 20X magnification with a Nikon Eclipse TI polarizing microscope. Digitized images were obtained using a Nikon DS Ri1 digital camera.

### Large Unilamellar Vesicles (LUVs) Preparation and Carboxyfluorescein Entrapment

The vesicles were prepared according to the reported protocol (Zhu et al., 2003; Williams et al., 2010). Briefly, the LUVs were prepared using three different lipids, DMPC, Cholesterol, and GM1 at 68:30:2 molar ratios and were solubilized to make 2 mM stock solution in chloroform and methanol (2:1) and the solvents were evaporated to make lipid films using nitrogen gas. The resulting lipid film was hydrated in 50 mM potassium phosphate buffer, pH 7.4 containing 5 mM carboxyfluorescein dyes. Then, the solution was vortexed vigorously for 30 min to emulsify the lipid mixtures. Next, the glass vial containing the lipid emulsion was dipped into liquid nitrogen for instant cooling and after 5 min the frozen solution was dipped into water bath at 50–60°C for thawing (Traïkia et al., 2000). This step was repeated five times. Excess dye was removed by ultracentrifugation, the supernatant dye solution was discarded, and the lipid pellet was re-hydrated with 50 mM potassium phosphate buffer. This step was repeated 2 more times and the final lipid pellet was collected followed by addition of 500 μL of potassium phosphate buffer and vortexed to obtain homogenous suspension of 2 mM of dye loaded LUVs. The dye leakage study was performed in triplicate on Infinite M200 microplate reader (Tecan, Switzerland). Excitation and emission wavelengths of carboxyfluorescein were 490 and 517 nm, respectively.

### Cell Cytotoxicity

The SH-SY5Y cell line (2 × 10^5^ cells/mL) was cultured in 96-well tissue microplates (100 μL/well) and allowed to adhere overnight at 37°C. The inhibitor molecules were dissolved in DMEM:Nutrient mixture F12 (Ham's) (1:1) (Biological Industries, Israel) at different concentrations (1, 5, 10, 20, 50, and 100 μM). The negative control, represented by zero, was prepared as medium without any hybrid molecules and treated in the same manner. 100 μL of medium with or without the inhibitor molecules were added to each well. Following incubation for 24 h at 37°C, cell viability was evaluated using the 2,3-bis(2-methoxy-4-nitro-5-sulfophenyl)-2H-tetrazolium-5-carboxanilide (XTT) cell proliferation assay kit (Biological Industries, Israel) according to the manufacturer's instructions. Briefly, 100 μL of the activation reagent was added to 5 mL of the XTT reagent, followed by the addition of 50 μL of activated-XTT solution to each well. After 2 h of incubation at 37°C, color intensity was measured using an ELISA microplate reader at 450 and 630 nm. Results are presented as mean and the standard error of the mean. Each experiment was repeated at least three times.

## Results and Discussions

Three conjugate molecules composed of Mannitol (**M**) chemically linked to NQTrp (**N**) were synthesized, either by click chemistry or via a PEG linker, termed as hybrid Mannitol-Click-NQTrp (**MCN**, Figure 1), Mannitol-2G-NQTrp (**M2N**, Figure 1), and Mannitol-3G-NQTrp (**M3N**, Figure 1: where 2/2G and 3/3G indicate two and three PEG unit, respectively). For details of the synthesis see Materials and Method section. We hypothesized that the chemical linkage (click or PEG) would not affect the individual activity of Mannitol or NQTrp or would have a negligible effect. The PEG linkers in hybrid molecules (**M2N** and **M3N**) were used to enhance the flexibility and biocompatibility (Harris and Chess, 2003).

### Kinetics of Inhibition of α-Syn Aggregation

**Figure 2:**
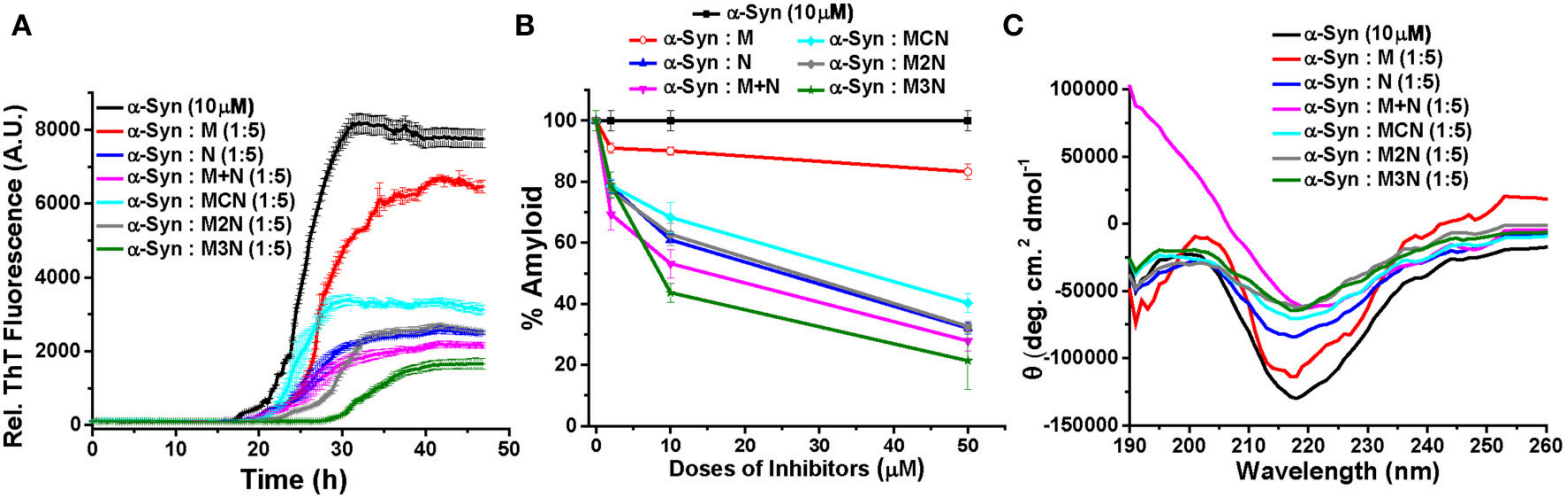
**(A)** Time dependent ThT fluorescence for the inhibition of α-Syn (10 μM) in absence (black curve) or presence of 5-molar excess of inhibitors, **M** (red curve), **N** (blue curve), (**M+N**) (magenta curve), **MCN** (cyan curve), **M2N** (gray curve), and **M3N** (green curve). **(B)** The end point ThT fluorescence represented as % amyloid remaining in the solution of α-Syn in absence or presence of different doses of the inhibitors. **(C)** CD spectra of α-Syn in absence (black curve) or presence of different doses of the inhibitors. CD Spectra were recorded after 50 h of incubation of α-Syn in the absence or presence of different doses of inhibitor molecules. All the experiments were performed in PBS (pH 7.4, 100 mM) at 37°C.

Mannitol only and NQTrp only (hereafter termed **M**, **N**, respectively) and an equimolar mixture of them (**M+N**) were used as reference. The aggregation kinetics of α-Syn in absence or presence of the different inhibitors was monitored by ThT fluorescence assay. ThT is an amyloid binding dye which fluoresces upon binding to the cross-β sheet structure of the amyloid fibrils. Its level of fluorescence is commonly used to monitor the amount of fibrils present in the solution (LeVine, 1999). α-Syn was dissolved in PBS buffer (pH 7.4, 100 mM) to obtained a final working concentration of 10 μM and was mixed with the inhibitors at various molar ratios (α-Syn : inhibitors = 5:1, 1:1, and 1:5). (Figures 2A,B, Figure S9).

The results reveled that α-Syn (10 μM) alone aggregates and forms fibrils after 30 h of incubation in PBS at 37°C as evident by the enhanced ThT fluorescence intensity with time (black curve, Figure 2A, Figure S9). All inhibitors significantly inhibited fibril formation of α-Syn in a dose dependent manner with greatest reduction of fluorescence intensity, indicating maximum inhibition of fibrillization of α-Syn, at 5-fold molar excess concentration of the various inhibitors (Figure 2A). However, even at 5-fold molar excess, Mannitol (**M**) could only inhibit aggregation by ~17% (red, Figure 2B), indicating that higher doses of Mannitol may be required for achieving substantial reduction of aggregation. This was in accordance with a previous report which found that to inhibit ~90% aggregation more than 2,000-fold molar excess of Mannitol was required (Shaltiel-Karyo et al., 2013). NQTrp (**N**, blue, Figure 2B) and the mixture of **M+N** (magenta, Figure 2B) inhibited α-Syn aggregation more efficiently than **M** (Figure 2B), yet inhibition by the mixture (**M+N**) was considerably more profound than NQTrp alone (73 vs. 68% inhibition with 1:5 molar ratio, respectively) at all molar ratios tested, suggesting a synergistic effect of Mannitol and NQTrp. When examining the effect of the three conjugates (**MCN**, **M2N**, **M3N**) on α-Syn aggregation it was evident that all of them display a dose dependent inhibition (cyan, gray, green respectively in Figure 2B). Interestingly, among the three conjugates, compound **M3N** showed the highest degree of inhibition (~79%) close to, and slightly higher than, the effect of the **M+N** mixture (~73%). This indicates that compound **M3N** retains the combined inhibitory property of compound **M** and **N**. Compound **M3N** reduced the extent of fibril formation as well as increased the lag time, which makes it more efficient among all the inhibitors tested. We speculate that the longer PEG in **M3N** than in **MCN** or **M2N** might confer upon its greater flexibility required for efficient inhibition than the other two conjugates where the shorter linker may be more rigid than required for efficient interaction with α-Syn.

### Conformational Transition of Inhibited α-Syn

The conformational transition of α-Syn in absence or presence of different doses of the various inhibitors was measured by Circular Dichroism (CD) at the end-point of fibril formation (Figure 2C, Figure S10). In the CD analysis α-Syn alone showed a negative band ~218 nm and a positive band ~198 nm, which were indicative of β-sheet rich conformation present in the fibrillar solution (black curve, Figure 2C, Figure S10) as reported (Shaltiel-Karyo et al., 2013). In the presence of various doses of Mannitol (**M**), the intensity of negative band at ~218 nm was slightly reduced (Figure S10A and red curve, Figure 2C), which indicates that 5-fold molar excess may not be high enough to reduce the β-sheet content of α-Syn significantly. In presence **N** (blue curve, Figure 2C), the β-sheet content of α-Syn was reduced in a dose dependent manner evidenced from the substantial reduction of CD intensity at 218 nm with increasing doses of **N** (Figure S10B). Likewise, a dose dependent effect was observed for the mixture (**M+N**) and all three conjugate molecules (**MCN**, **M2N**, and **M3N**, Figures S10D–F).

### Morphology of Inhibited α-Syn Assemblies

**Figure 3:**
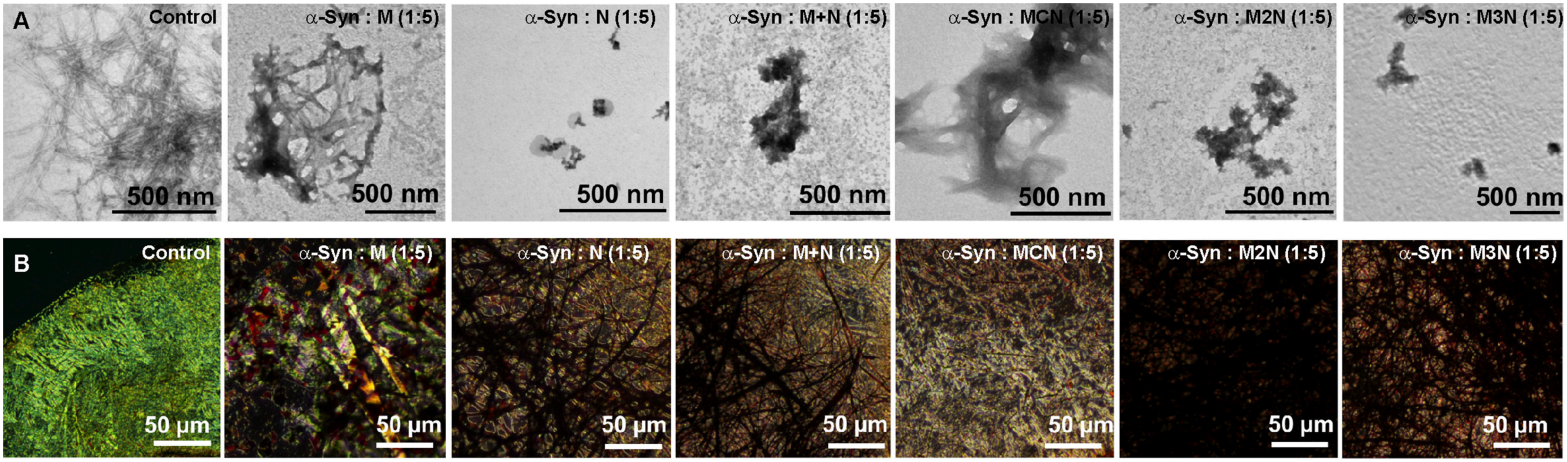
**(A)** TEM images and **(B)** Congo red stained birefringence images of α-Syn (10 μM) in absence and presence of 1:5 molar ratio (α-Syn: inhibitor) of the inhibitor molecules. Images were captured after 50 h of incubation of α-Syn in absence and presence of the inhibitor molecules in PBS (pH 7.4) at 37°C.

Morphology of α-Syn assemblies in absence and presence of the inhibitors was examined at the end-point of the fibrillization kinetics by transmission electron microscopy (TEM) (Figure 3A, Figure S11A). Since the ThT and CD analyses indicated that at 5:1 molar ratio of α-Syn:inhibitors there was hardly reduction of the fibrillization, TEM analysis was performed with 1:1 and 1:5 molar ratio. Assemblies of α-Syn alone appeared as dense fibrillar aggregates (Figure 3A) as reported (Scherzer-Attali et al., 2012). At equimolar ratio (1:1) doses of the inhibitors **M**, **N**, (**M+N**), and **MCN**, the fibrillar aggregates of α-Syn remained visible, indicating that the dose was not high enough to reduce the level of aggregates significantly (Figure S11). However, the conjugate inhibitors **M2N** and **M3N** at equimolar markedly reduced the fibrillar assemblies (Figures S11vi,vii). In presence of 5-fold molar excess of **N**, (**M+N**), **M2N**, and **M3N**, the density of fibrillar assemblies was significantly reduced. However, a mixture of amorphous and fibrils was observed in presence of 5-fold molar excess of inhibitor **MCN** (Figure 3A), and no reduction of fibrils was observed in presence 5-fold molar excess of Mannitol (**M**), which supports the ThT results (Figure 3A).

### Congo Red Birefringence of Inhibited α-Syn Assemblies

Congo red is known as an amyloid specific dye and exhibits yellow green birefringence upon binding with ordered amyloid structures under cross polarized light (Westermark et al., 1999). We used it to further validate the effect of the inhibitors on α-Syn fibrillization. Following incubation of α-Syn (10 μM) in the absence of inhibitors for 50 h Cong red staining resulted in a clear green-gold or apple-green birefringence, indicating the presence of amyloid fibrils in the sample (Figure 3B, Figure S11Bi). Some green-gold birefringence was observed in α-Syn samples that were incubated with 1:1 molar ratio of the inhibitors, indicating that 1:1 molar ratio was not sufficient for inhibition of α-Syn fibrillar aggregates, except compound **M2N** and **M3N** (Figure S11B) which were more effective, as observed in morphological analysis by TEM. In contrast, no birefringence was observed when α-Syn was treated with 1:5 molar ratio of all inhibitors tested except in presence of Mannitol (**M**) (Figure 3B). Collectively, the results of ThT, CD, TEM, and Congo red birefringence, indicate that the conjugate molecule **M3N** was a superior among all tested molecules.

### Effect of the Inhibitors on α-Syn Cytotoxicity

α-Syn assemblies are believed to exert their neurotoxicity in PD by damaging the cell membrane (Winner et al., 2011; Xin et al., 2015; Ghiglieri et al., 2018). To evaluate whether the tested molecules can reduce toxicity of α-Syn oligomers and fibrils, we employed two complementary assays, one involving Large Unilamellar Vesicles (LUVs) and the other involving cultured neuronal cells. Monitoring the extent of leakage of Carboxyfluorescein dye from LUVs loaded with it is a commonly used proxy for damage to cell membrane and cytotoxicity by amyloids only or is this a general assay for membrane leakage (Zhu et al., 2003; Williams et al., 2010; Fecchio et al., 2013).

Prior to the leakage assay, the formation of LUVs was confirmed by TEM imaging and they were found to be ~200 nm in diameter with a characteristic spherical morphology (Figure S12). As a reference Carboxyfluorescein leakage from dye loaded LUVs treated with 10% Triton X-100, was consider as 100%. Leakage value was calculated from the following equation (McLaurin and Chakrabartty, 1996):

$$\%\;dye\;leakage=\;\frac{Fluorescence_{observed}-Fluorescence_{initial}}{Fluorescence_{total}-Fluorescence_{initial}}\;\times 100$$

**Figure 4:**
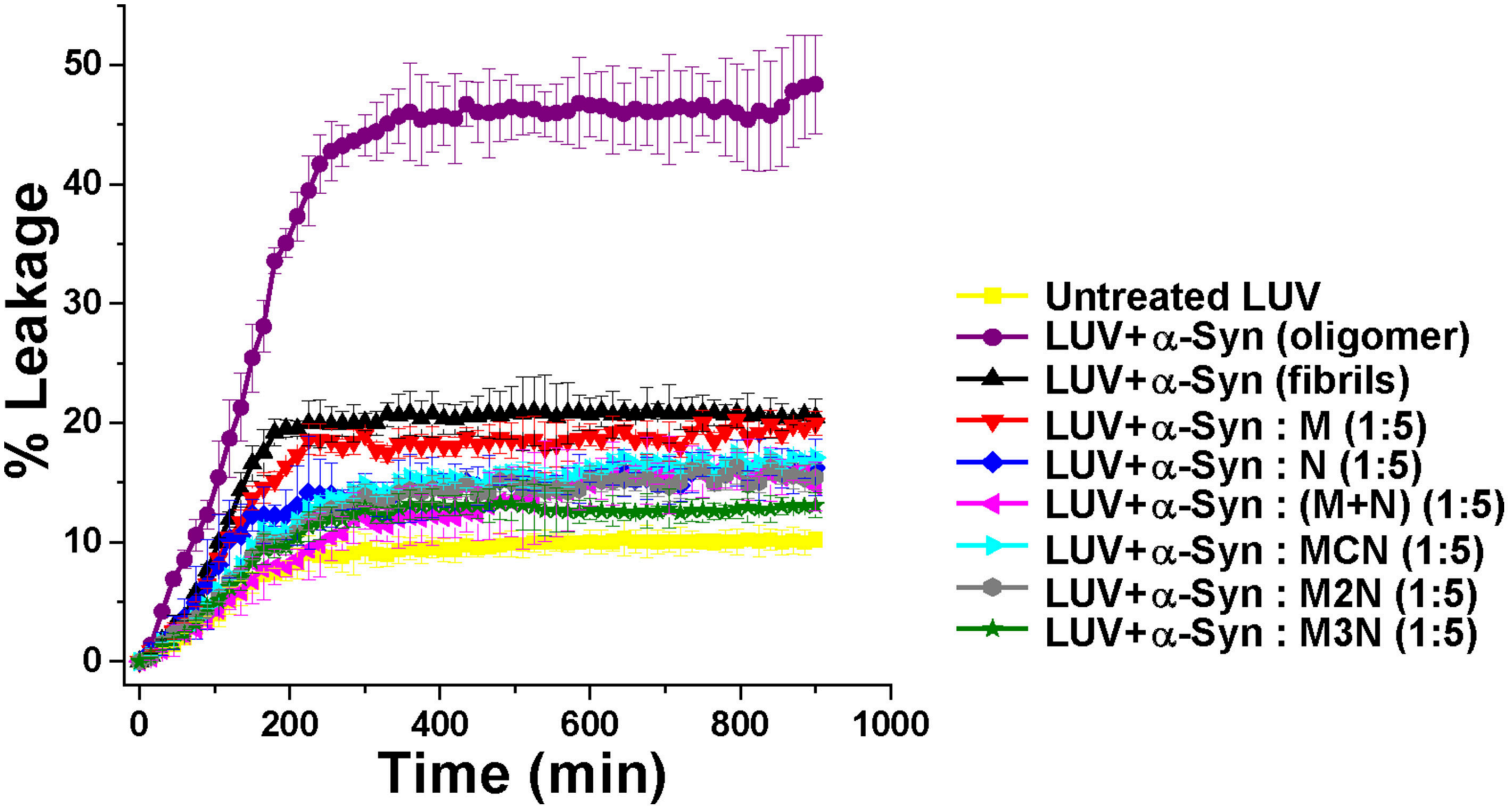
Percentage of dye leakage with time from LUVs treated with α-Syn samples in absence or presence of 5-fold molar excess of the inhibitor molecules. The leakage from LUVs treated with Triton X-100 was set as 100%. Carboxyfluorecein was excited at 490 nm and emission was recorded at 517 nm.

Based on that, spontaneous dye release from the untreated LUVs was calculated as 8% (yellow curve, Figure 4). For the control experiment, in absence of inhibitors, α-Syn oligomers or fibrils were generated by incubating α-Syn monomers for 25 or 50 h respectively (based on the ThT results in Figure 2A). Incubation of α-Syn oligomers in absence of inhibitors with the dye-loaded LUVs, resulted in high leakage reaching ~ 46% after 600 min (purple curve, Figure 4). In contrast, mature α-Syn fibrils caused only 16% of dye leakage (black curve, Figure 4). For evaluating the effect of the inhibitors on α-Syn induced LUV leakage, α-Syn monomers were incubated with 5-fold molar excess of the inhibitor molecules for 50 h, during which both oligomers and fibrils could have been formed. Next, the samples were each mixed with the dye-loaded LUVs and leakage was monitored for 900 min in 10 min interval.

The samples of α-Syn with the inhibitor molecules caused lower level of leakage in comparison to α-Syn oligomers without inhibitors. Least amount of leakage (~11%) was observed for the α-Syn sample in presence of 1:5 molar ratio of compound **M3N** (green curve, Figure 4), indicating that the molecule **M3N** prevented formation of membrane disrupting species of α-Syn.

To compare the level of leakage caused by α-Syn in the presence of 5-fold molar excess of (**M+N**) and **M3N** at different time points, we performed an additional LUV leakage assay by incubating α-Syn monomers in absence or presence of these inhibitors for 25 and 50 h (Figure S13). In the absence of the inhibitors we observed that 25 h aged α-Syn assemblies, which were considered as oligomers, caused higher level of dye leakage (~43%) in comparison to mature fibrils (aged 50 h) which caused lesser dye leakage (~16%) (Figure S13B). In contrast, in the presence of 5-fold molar excess of (**M+N**) and **M3N**, the 25 h aged α-Syn caused lesser dye leakage (~19% and ~14%, respectively) than in their absence (~43%). Likewise, for 50 h aged α-Syn (fibrils) 5-fold molar excess of (**M+N**), and **M3N** caused lesser dye leakage (~13 and ~10%, respectively) than in their absence (~16%) (Figures S13A,B). Combining the results of dye leakage by α-Syn at different incubation times in the absence or presence of these inhibitors, we observed that **M3N** caused lesser dye leakage at the different time points of α-Syn aggregation than **M+N**, supporting the conclusion that the conjugate molecule **M3N** impeded formation of membrane disrupting species of α-Syn.

**Figure 5:**
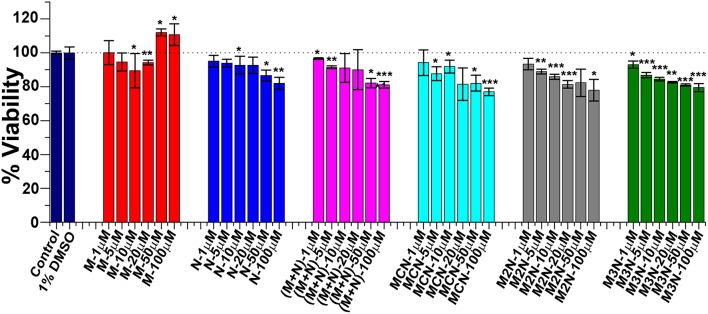
Evaluation of cytotoxicity of the inhibitor molecules toward SH-SY5Y cells. Cells were incubated with the inhibitors at different concentrations (1–100 μM) for 24 h and cytotoxicity was measured by XTT assay. One percent DMSO was used to solubilize the tested compounds (used as vehicle). Untreated cells were used as control and set to 100% viability. Results are average of 3 independent assays (*n* = 3–6, ±SD) and are expressed as percentage of control cells. Significance (^*^p < 0.05), (^**^p < 0.005), and (^***^p < 0.001).

In preparation for evaluating the effect of the various inhibitors on α-Syn toxicity toward cells, we first examined whether the inhibitors themselves exhibited cytotoxicity. SH-SY5Y neuroblastoma cells were incubated with the various inhibitors were incubated at different concentrations for 24 h and the viability of cells were analyzed by XTT reduction assay (Figure 5). No substantial cytotoxic effects of the inhibitors themselves on these cells was observed, even at the highest concentration of 100 μM.

**Figure 6:**
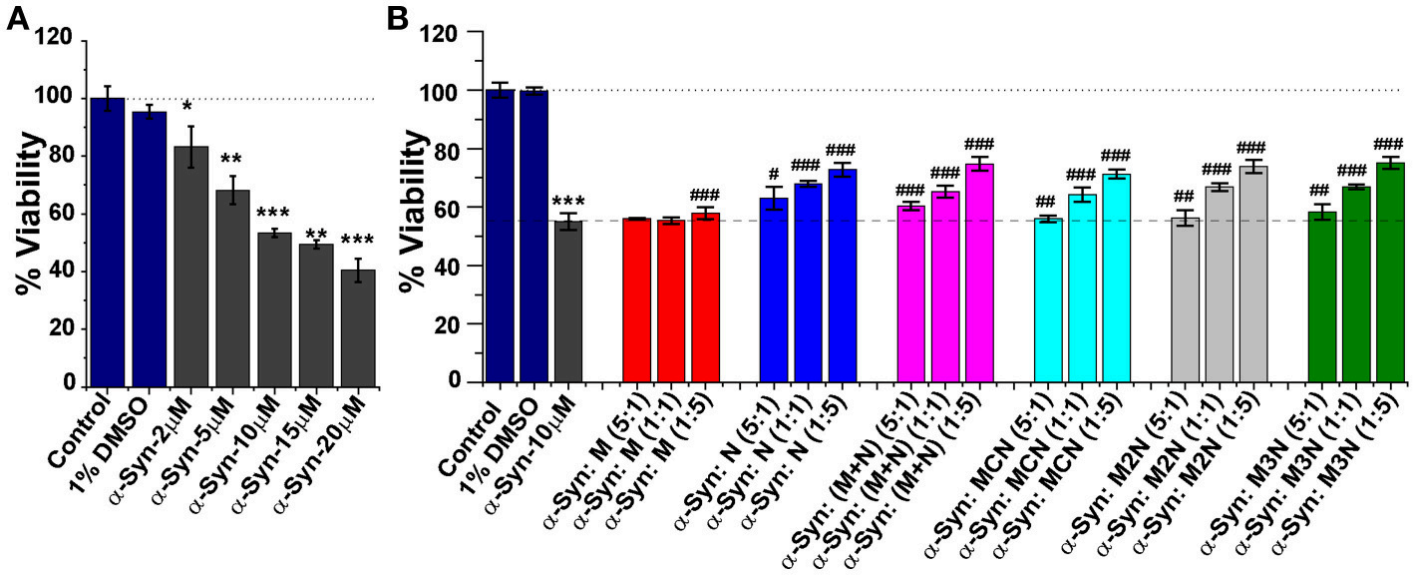
**(A)** Relative viability of SH-SY5Y cells in presence of different concentrations (2–20 μM) of α-Syn aggregates measured by XTT assay. **(B)** Dose-dependent effect of the inhibitor molecules on α-Syn mediated cytotoxicity toward SH-SY5Y cells. α-Syn (10 μM) monomers were incubated in absence or presence of different doses of the inhibitor molecules (5:1, 1:1, and 1:5) for 50 h and was applied to the SH-SY5Y cells for additional 24 h. Cell viability was measured by XTT assay, Untreated cells were used as control and set to 100% viability. Results are average of 3 independent assays (*n* = 3–6, ±SD) and are expressed as percentage of control cells. ^*^p < 0.05, ^**^p < 0.005, and ^***^p < 0.001 compared to control group. ^**#**^*p* < 0.05, ^**##**^*p* < 0.005, and ^**###**^*p* < 0.001 compared to α-Syn treated group.

α-Syn monomers (2–20 μM) were allowed to preaggregate for 50 h. When incubated with SH-SY5Y for 24 h a dose dependent reduction of viability of the cells was observed with an IC_50_ value (50% cell death) of ~11 μM (Figure 6A). To evaluate the effect of the inhibitor molecules on cytotoxicity induced by α-Syn aggregates induced cytotoxicity, α-Syn (10 μM) was allowed to aggregate for 50 h in the absence or presence of various molar ratios (α-Syn : inhibitor = 5:1, 1:1, 1:5) of the inhibitors. Next, these samples were incubated with the cells for additional 24 h followed by XTT viability assay.

As shown in Figure 6B, the inhibitor molecules significantly attenuated the cytotoxic effect of α-Syn aggregates in a dose dependent manner evidenced as increase of cell viability. At lower dose [0.2 molar excess (5:1)] none of the inhibitors could increase cell viability. Significant increment in viability was observed with increased doses of the inhibitors. For example, at 5-fold molar excess of the inhibitors, compound **N** increased cell viability up to ~73%, (**M+N**) 75%, **MCN** 72%, **M2N** 73%, and **M3N** 75%. Mannitol (**M**) had a marginal effect on α-Syn induced cytotoxicity, which agrees with the ThT, TEM, and LUV results.

## Conclusions

Our systematic study of the aggregation α-Syn and its inhibition by the Mannitol-NQTrp based conjugate molecules revealed that all the tested molecules including the controls (**M**, **N**, and **M+N**) efficiently slowed down the aggregation kinetics of α-Syn in a dose dependent manner. Mannitol (**M**) was the least effective among the tested molecules as evident both from ThT, CD, and TEM assays and this was reflected in its low effect on α-Syn cytotoxicity. NQTrp (**N**) was more effective, yet the mixture (**M+N**) displayed higher efficiency than **N**, indicating synergistic effect of Mannitol and NQTrp. Among the novel conjugate molecules compound, **M3N** was the most effective in delaying the process of fibrillization as well as most significantly reduced the β-sheet content and the extent of fibrils formed. The highest efficacy of **M3N** was also manifested in its strongest ability to attenuate membrane disruption by α-Syn. **M3N** was not significantly better in preventing cytotoxicity of α-Syn aggregates than **N** or **M+N** Conjugate **M3N** appears to have similar or better capabilities as (**M+N**) mixture, indicating that by employing a longer linker it retained the beneficial attributes of both Mannitol and NQTrp. Since **M3N** also has low cytotoxicity by itself, it can be attractive as a potential scaffold for development of therapeutic molecules for PD.

## Author Contributions

AP, Y-ML, and DS conceived the project. AP performed all the experiments and analyzed the data. B-DZ synthesized all the designed molecules. SM performed Congo red birefringence assay. GL characterized all the molecules synthesized. AP and DS wrote the manuscript with the help of Y-ML and EG. All authors read and approved the manuscript.

### Conflict of Interest Statement

The authors declare that the research was conducted in the absence of any commercial or financial relationships that could be construed as a potential conflict of interest.
