# Supplementary material for: Novel Mannitol-Based Small Molecules for Inhibiting Aggregation of α-Synuclein Amyloids in Parkinson's Disease
Source: Front Mol Biosci. 2019 Mar 22;6:16. doi: 10.3389/fmolb.2019.00016 (PMC6438916; doi:10.3389/fmolb.2019.00016)
Supplement: Supplementary file 1 [file Table_1.docx]

Supplementary Materials

**Novel Mannitol-Based Small Molecules for Inhibiting Aggregation of α-Synuclein Amyloids in Parkinson’s Disease**

Ashim Paul^1^, Bo-Dou Zhang^2^, Satabdee Mohapatra^1^, Gao Li^2^, Yan-Mei Li^2,3,4^, Ehud Gazit^1^, and Daniel Segal^1,5*^

^1^School of Molecular Microbiology & Biotechnology, Tel Aviv University, Tel Aviv 69978, Israel

^2^Department of Chemistry, Tsinghua University, Beijing 100084, China

^3^Beijing Institute for Brain Disorders, Beijing 100069, China

^4^Center for Synthetic and Systems Biology, Tsinghua University, Beijing 100084, China

^5^Sagol Interdisciplinary School of Neurosciences, Tel Aviv University, Tel Aviv 69978, Israel

*** Correspondence:**

Prof. Daniel Segal

[dsegal@post.tau.ac.il](mailto:dsegal@post.tau.ac.il)

**Supplemental Schemes**

**Scheme S1:** A schematic representation for stepwise synthesis of compound **MCN**.

**Scheme S2:** A schematic representation for stepwise synthesis of compound **M2N** and **M3M**. In the scheme, n=2, represents the compound **M2N** and n=3 represent compound **M3N**.

.

**Supplemental Figures**

**
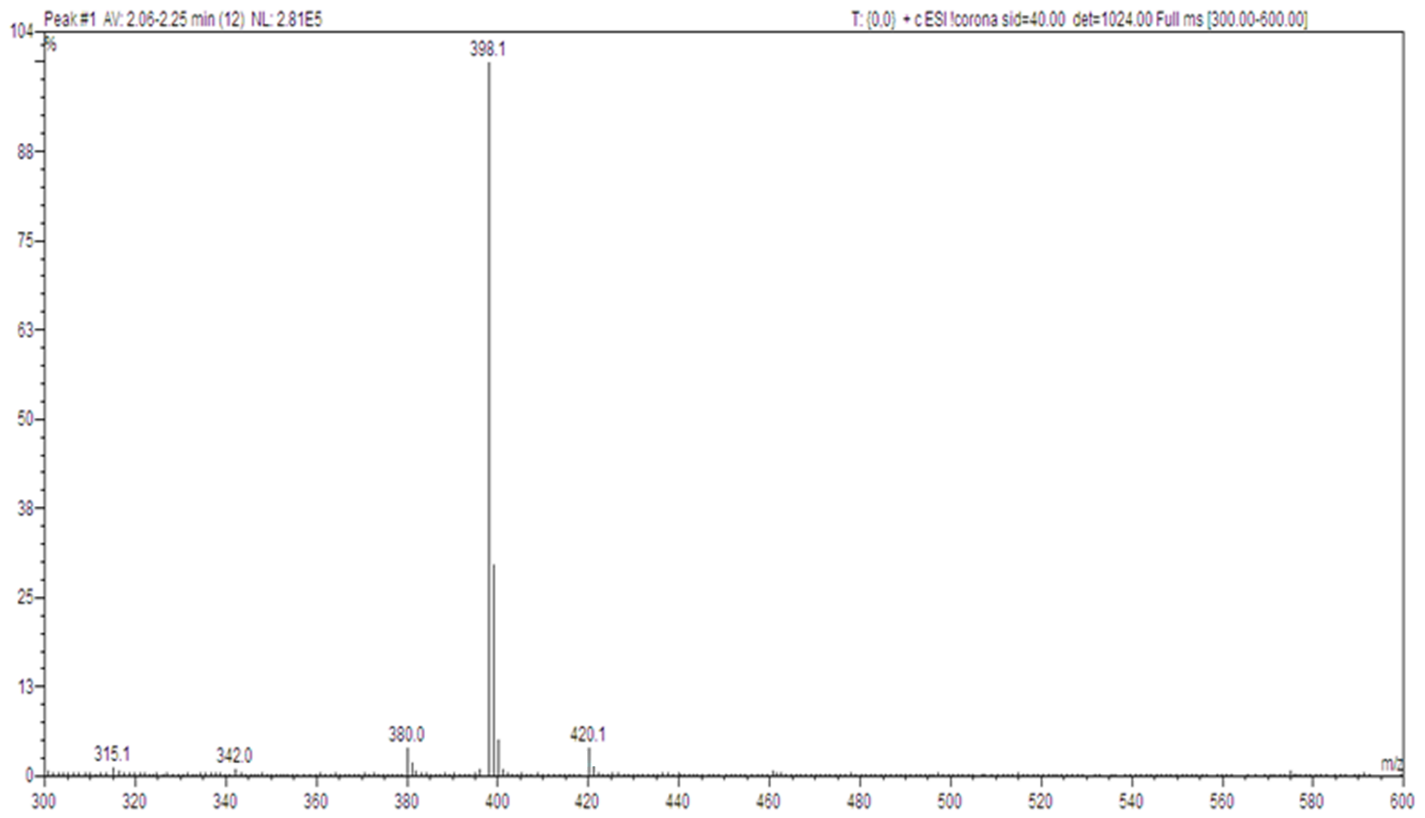
**

**Figure S1:** Mass spectrum of Compound **2** in Scheme S1. Calculated mass for C_24_H_20_N_3_O_3_ is 398.1 [M+H]^+^, observed 398.1 [M+H]^+^.


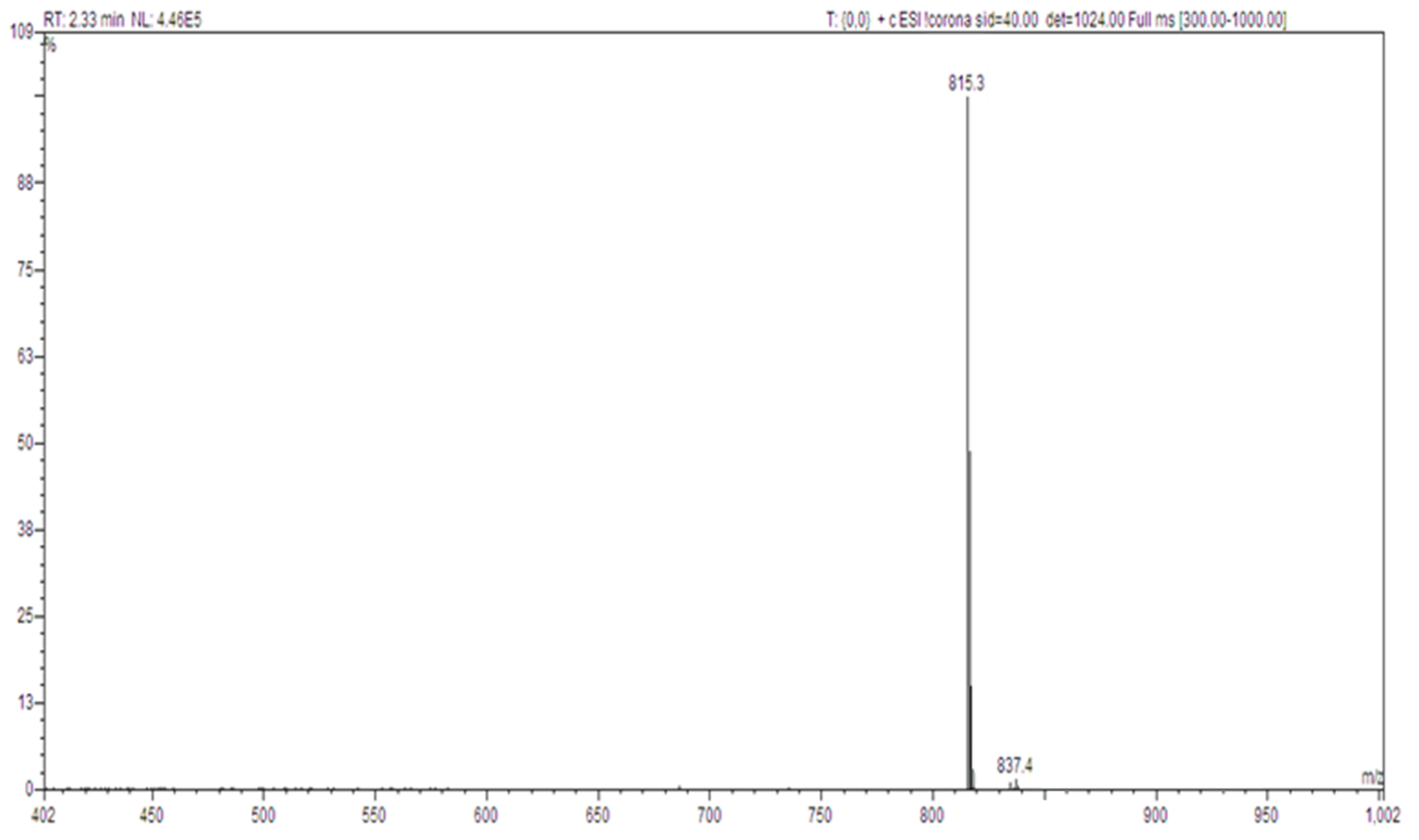


**Figure S2:** Mass spectrum of Compound **7** in Scheme S1. Calculated mass for C_40_H_43_N_6_O_13_ is 815.3 [M+H]^+^, observed 815.3 [M+H]^+^.

**Figure S3:** HPLC profile of the purified Compound **MCN**.


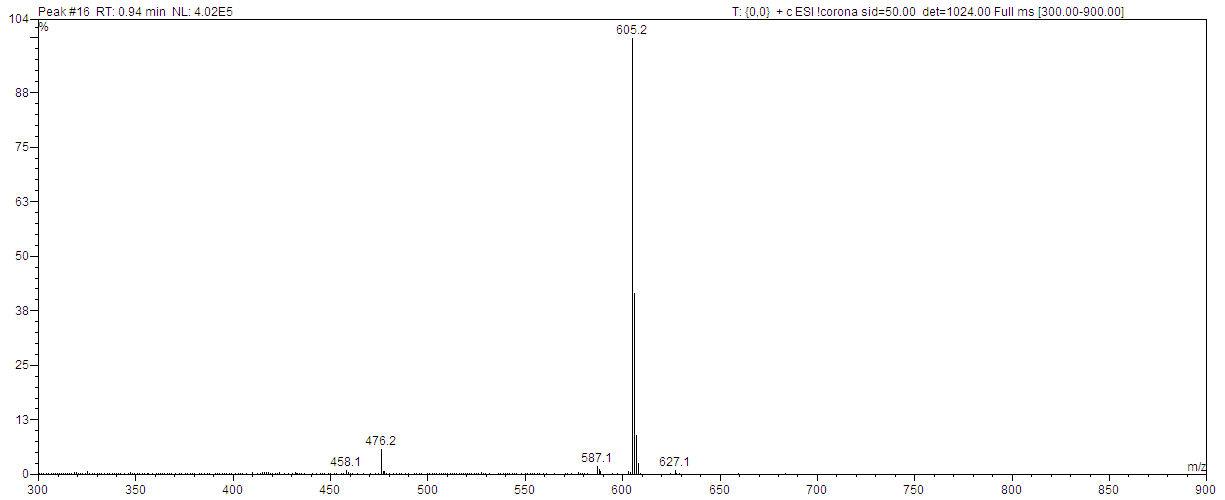


**Figure S4:** Mass spectrum of Compound **MCN**. Calculated mass for C_30_H_33_N_6_O_8_ is 605.2 [M+H]^+^, observed 605.2 [M+H]^+^.

**Figure S5:** HPLC profile of the purified Compound **M2N**.


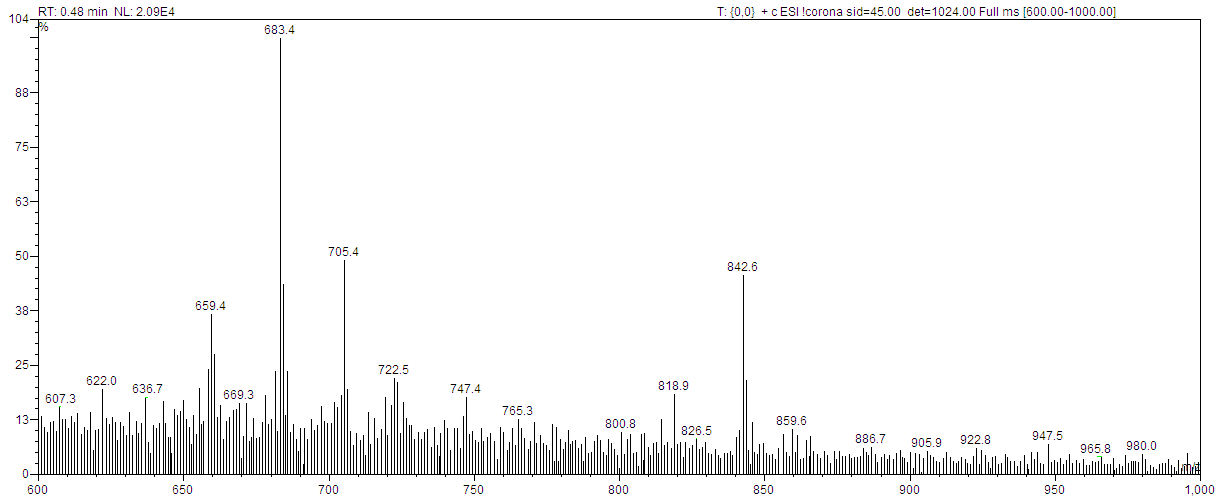


**Figure S6:** Mass spectrum of Compound **M2N**. Calculated mass for C_34_H_43_N_4_O_11_ is 683.3 [M+H]^+^, observed 683.4 [M+H]^+^.

**Figure S7:** HPLC profile of the purified Compound **M3N**.


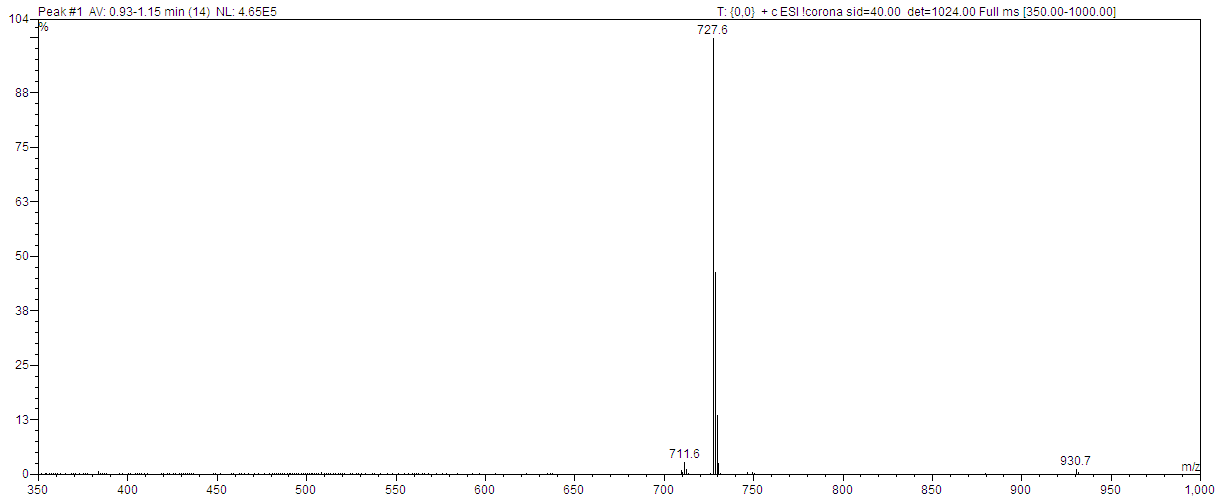


**Figure S8:** Mass spectrum of Compound **M3N**. Calculated mass for C_36_H_47_N_4_O_12_ is 727.3 [M+H]^+^, observed 727.6 [M+H]^+^.


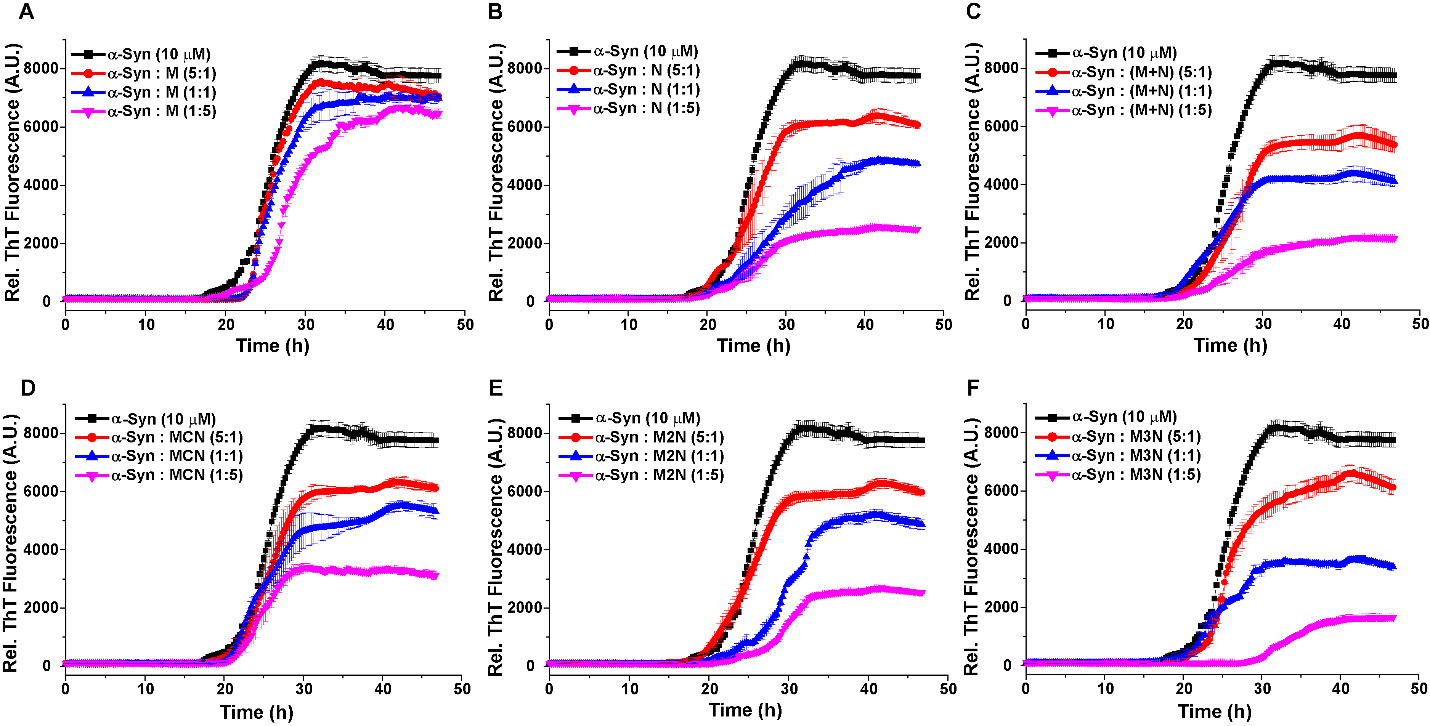


**Figure S9:** Time dependent ThT fluorescence for the inhibition of α-Syn in absence (black curve, A-F) or presence of different doses of compound **M** **(A)**, compound **N** **(B)**, mixture (**M+N**) **(C)**, conjugate **MCN** **(D)**, conjugate **M2N** **(E)** and conjugate **M3N** **(F)**. Experiment were performed in PBS (pH 7.4) at 37 ºC.


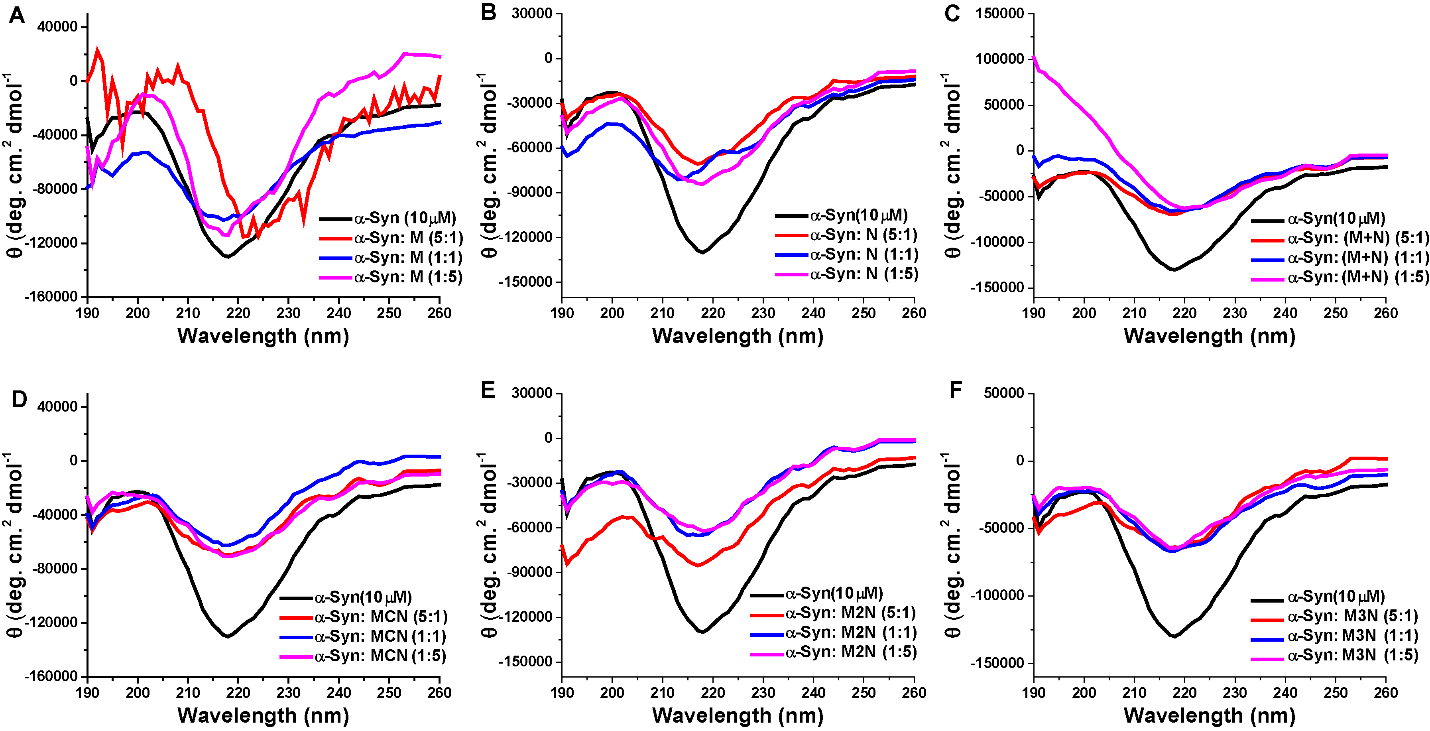


**Figure S10:** CD spectra of α-Syn in absence (black curve, A-F) or presence of different doses of compound **M** **(A)**, compound **N** **(B)**, mixture (**M+N**) **(C)**, conjugate **MCN** **(D)**, conjugate **M2N** **(E)** and conjugate **M3N** **(F)**. Spectra were recorded after 50h of incubation of α-Syn in the absence or presence of different doses of the inhibitor molecules in PBS (pH 7.4) at 37 ºC.


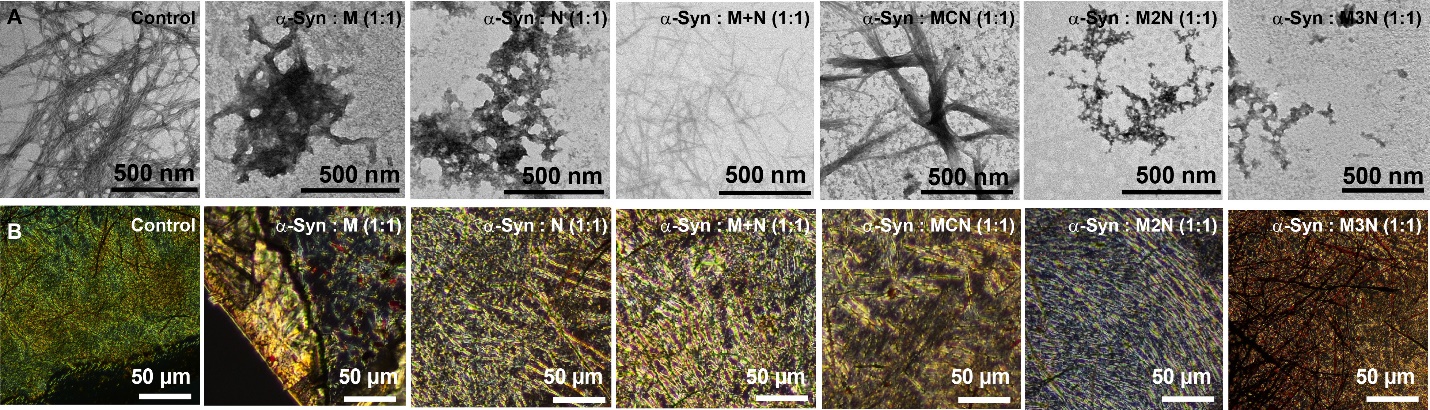


**Figure S11:** **(A)** TEM images and **(B)** Congo red stained birefringence images of α-Syn in absence or presence of 1:5 molar ratio (α-Syn : inhibitor) of the inhibitor molecules. Images were captured after 50 h of incubation of α-Syn in absence or presence of equimolar ratio of the inhibitor molecules in PBS (pH 7.4) at 37 ºC.


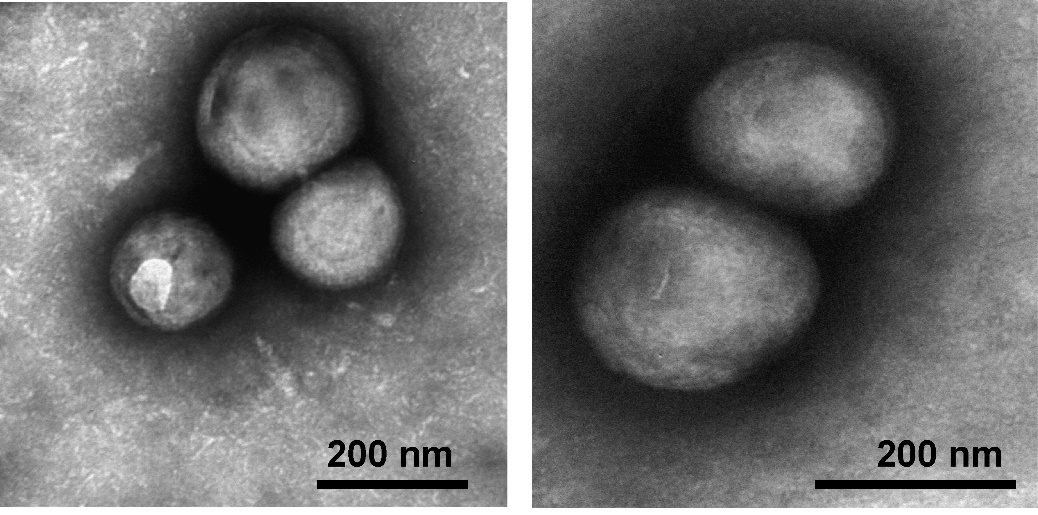


**Figure S12:** TEM images of the prepared LUVs.


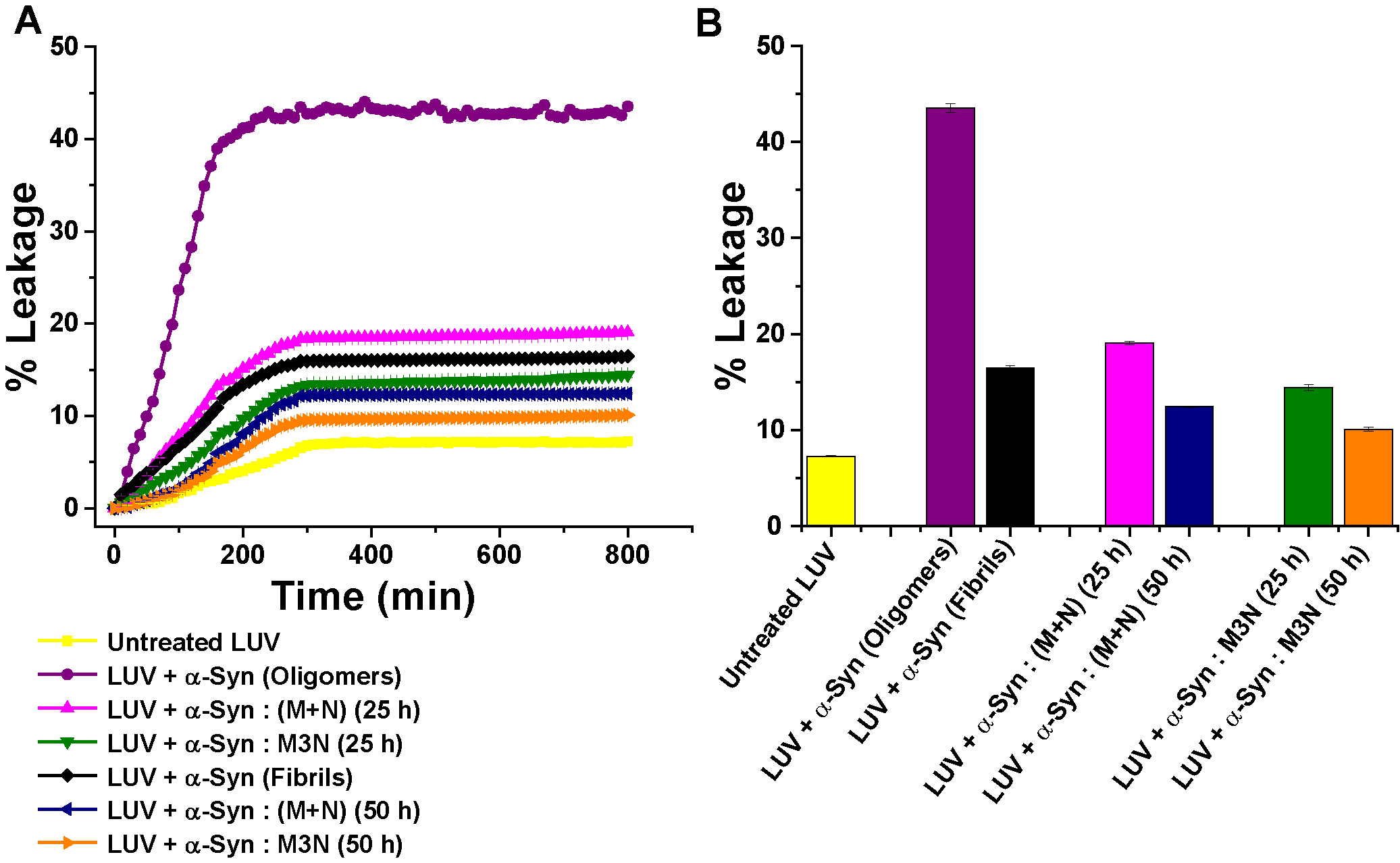


**Figure S13:** **(A)** Percentage of dye leakage with time from LUVs treated with 25 h or 50 h aged α-Syn samples in absence or presence of 5-fold molar excess of (**M+N**) and **M3N**. **(B)** Bar-diagram showing % dye leakage from LUVs treated with α-Syn samples in absence and presence of 5-fold molar excess of (**M+N**) and **M3N** at the end point of the leakage assay. The leakage from LUVs treated with Triton X-100 was set as 100%. Carboxyfluorecein was excited at 490 nm and emission was recorded at 517 nm.
